# Effective and Efficient Pretreatment of Polyimide Substrates by Capacitively Coupled Plasma for Coating the Composites of Tetracycline-Imprinted Polymers and Quantum Dots: Comparison with Chemical Pretreatment

**DOI:** 10.3390/s20092723

**Published:** 2020-05-10

**Authors:** Ching-Bin Ke, Jian-Lian Chen

**Affiliations:** 1 Department of Beauty and Health Care, Min-Hwei Junior College of Health Care Management, No.1116, Sec 2, Zhongshan E. Rd., Tainan 73658, Taiwan; A262@o365.mhchcm.edu.tw; 2 School of Pharmacy, China Medical University, No. 91 Hsueh-Shih Road, Taichung 40402, Taiwan

**Keywords:** fluorescence, imprinted polymer, plasma, polyimide, quantum dot, tetracycline

## Abstract

Composites of tetracycline (Tc)-imprinted polymethacrylates and quantum dots have been coated on chemically pretreated polyimide substrates (PIs) as fluorescent sensors. In this study, PIs were pretreated by capacitively coupled plasma (CCP) before coating the same composites on them. For the first time, to fabricate sensors by plasma modification of PIs, the CCP conditions, including plasma gas, flow rate, radio frequency generation power, and duration time, the fabrication details, including coating, baking, and stripping steps, and the sample loading process were optimized to perform a linear decrease in fluorescent intensity with Tc concentrations in the range of 5.0–3000 μM (R^2^ = 0.9995) with a limit of detection of 0.2 μM (S/N = 3, relative standard deviation (RSD) = 2.2%). The selectivity of the stripped PIs was evaluated by the imprinting factors (IFs) for Tc (IF = 7.2), other Tc analogues (IF = 3.4–5.3), and steroids (IF ≈ 1) and by the recoveries of 5.0 μM Tc from bovine serum albumin at 300 μg∙mL^−1^ (98%, RSD = 3.2%), fetal bovine serum at 1.5 ppt (98%, RSD = 2.8%), and liquid milk (94.5%, RSD = 5.3%). The superiority of the present plasma-treated-based sensor over the previous chemically-treated one in fabrication efficiency and detection effectiveness was clear.

## 1. Introduction

Plastic substrates have been successfully used in electrochemical sensors with advanced sensing materials and fabrication techniques, such as substrate pretreatment, film deposition, line patterning, and module assembly, at low temperatures that a plastic substrate can sustain [1,2,3]. Many electrochemical sensors are electronic noses, which aim at gas detection to avert false conductive connections between the sensing elements in solution. In contrast to electrochemical sensors, optical sensors are built less commonly on plastic substrates and are mostly applied to aqueous bioassays. Fluorometric assays prevail over colorimetric assays, which are restricted to transparent substrates [4], and are immobilized with antibodies [5,6], cells [7], and peptide [8] via passive adsorption [5], photo-cross-linkable hydrogels [7], and covalent bonding [6,8], respectively, on the plastic surfaces to detect fluorophore-labeled targets.

Polyimide (PI) is one of the materials most used as a plastic substrate. For improving the adhesion of coating materials on the chemically inert PI substrate, electron beam [9], laser [10], ion beam [11], ultraviolet (UV)/O_3_ radiation [12], gamma-ray [13], plasma [14,15,16,17,18], and chemicals [19,20,21], have been applied to PI surfaces before coating. In contrast with the dry treatments, wet chemical modification possesses lower instrument expenses but more tedious procedures. Plasma is the most prevalent process among the dry-type options. For example, O_2_ [14,15], O_2_/CF_4_ [16], Ar [17], and Ar/H_2_ [18] plasmas are favorable for Ag [14], ZnO [15], Ti/Cu [16], Al [17], and Al/Nd_2_Zr_2_O_7_ [18] coatings. In addition to a few studies on precursor film deposition [22,23], physical ion bombardments, which create hydrophilicity and roughness on PI surfaces, are used to enhance the peel strength of metals and metal composites. To the best of our knowledge, sensors fabricated with dry modification of PIs have not been described to date, although chemical modification of PIs has been used as a substrate for bonding quantum dots (QDs) as fluorescent sensors in our previous study [21].

QDs are fluorescent semiconductor nanocrystals and are often applied in sensing and bioimaging [24,25]. The conjugation of recognition receptors, such as complexometric reagents [26], cyclodextrins [27], antibodies [28], aptamers [29], and molecularly imprinted polymers (MIPs) [30,31], with QDs is crucial to develop QD-based fluorescent sensors. Among the receptors, MIPs uniquely provide versatile imprinted cavities with specific three-dimensional binding sites to recognize the complementary template molecules. Compared to the dispersion of MIP-QD composites in aqueous solutions, the deposition of the MIP-QDs on solid substrates, e.g., on quartz [32], glass [33,34], or PI [21], is rarely investigated.

In this study, PI substrates were treated by capacitively coupled plasma and their efficiency and effectiveness in sensor fabrication and fluorescence measurement were compared with chemically treated PI [21] and glass [34] substrates as they were all coated with the same composite of tetracycline (Tc)-imprinted polymethacrylate and CdTe QD after the treatments. As the optimization of the plasma recipe, the changes in surface free energy and the Fourier transform infrared (FTIR) spectrum of the treated PI substrates were analyzed and correlated with fluorescence performance. Other significant steps in subsequent processes, including coating the MIP-QD composites on the treated PIs, stripping the Tc from the MIP-QD composites, and sensing the Tc through a fluorescence-quenching mechanism, were also optimized and noticed with the difference between the studies. The sensitivity in the dose-response, stability in replicate measurement and storage, and selectivity in imprinting factor and recoveries from bovine serum albumin, fetal bovine serum, and fresh liquid milk, were investigated for the studied sensor.

## 2. Materials and Methods

### 2.1. Chemicals and Materials

Ethanol, 1,4-butanediol, 1-propanol, and phosphate salts were purchased from Acros (Thermo Fisher Scientific, Geel, Belgium). Allyl mercaptan (AM), methacrylic acid (MAA), and trichloroacetic acid were purchased from Alfa Aesar (Ward Hill, MA, USA). Ethylene glycol dimethacrylate (EGDMA) and α,αʹ-azobisisobutyronitrile (AIBN) were purchased from Aldrich (Milwaukee, WI, USA). Five tetracycline antibiotics, including tetracycline (Tc), oxytetracycline, doxycycline, minocycline, and methacycline were purchased from Sigma-Aldrich (Milwaukee, WI, USA). Steroids, including β-estradiol, cholic acid, and hydocortisone, bovine serum albumin (BSA, lyophilized powder, mol. wt. ~66 kDa), and fetal bovine serum (FBS, liquid, hemoglobin ≤ 25 mg∙dL^−1^) were purchased from Sigma (Milwaukee, WI, USA). Purified water (18 MΩ∙cm) from a Milli-Q water purification system (Millipore, Bedford, MA, USA) was used to prepare the standard salt and buffers. All standard solutions were protected from light and kept at 4 °C in a refrigerator. Sheets of polyimide (copolymer of pyromellitic dianhydride and oxydianiline (PMDA–ODA), A4 size, 175 μm thick) were purchased from Lih Kuang Industry Co., Ltd. (Taichung, Taiwan).

### 2.2. Synthesis of Molecularly Imprinted Polymers with Quantum Dots (MIP-QD) Composites Coated on Plasma-Treated Polyimide (PI) Substrates 

#### 2.2.1. Preparation of the MIP-QDs

**Figure 1:**
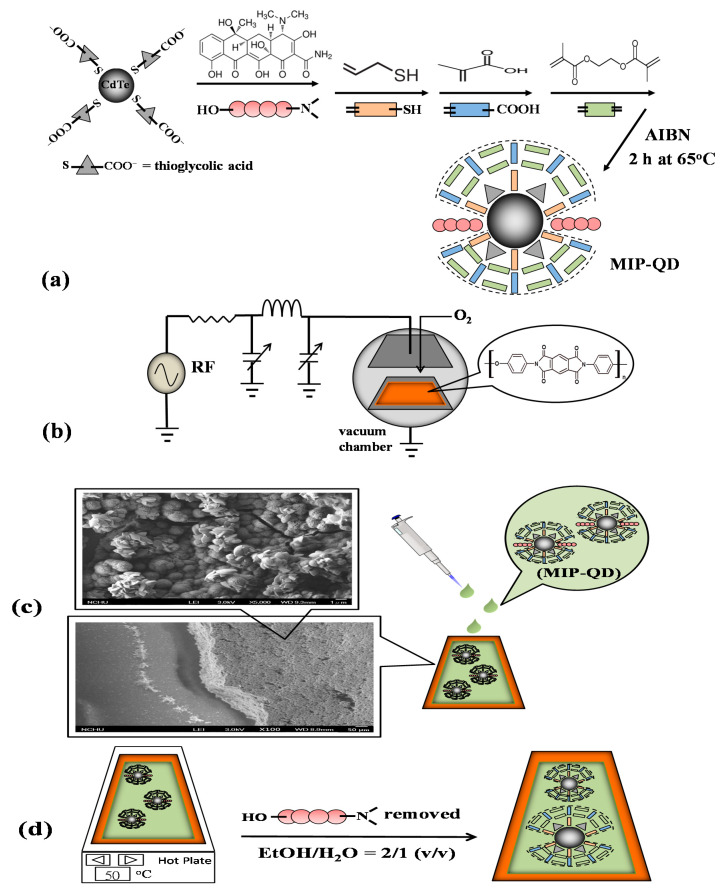
Schemes to (**a**) synthesize molecularly imprinted polymers with quantum dots (MIP-QD) composites, (**b**) pretreat polyimide substrates (PIs) with capacitively coupled plasma (CCP), (**c**) coat the MIP-QDs on the plasma-treated PIs, and (**d**) strip the tetracycline (Tc) templates from the PIs. Scanning electron microscope (SEM) images of the cross-section and a top surface of the complete MIP-plasma-PI are inserted in (**c**).

The recipe for the preparation of the methacrylic MIP-QD solution schemes in Figure 1a [21]. In brief, 2 mmol of MAA, 2 mmol of EGDMA, 1.2 mL of porogen composed of 1,4-butanediol, 1-propanol, and water in 1:1:1 ratio, 2 mmol of AM, 750 μL of the CdTe solution [30,31], 5.0 mg of Tc in 200 μL EtOH, 3.0 mL of EtOH, and 50 mg of AIBN in 500 μL were sequentially mixed in a flask filling with nitrogen, and then heated at 60 °C for two and half hours. The resultant MIP-QD solution would be coated on the plasma-treated PI substrates. If Tc was not involved in the mixing, the resultant solution was a contrastive MIP-QD one, i.e., non-imprinted polymer (NIP) conjugated with QDs (NIP-QDs).

#### 2.2.2. PI Substrates Treated with Capacitively Coupled Plasma and Coated with MIP-QDs

The received PI sheets were cut into pieces (2.5 cm × 4.0 cm), cleaned with ethanol, and dried in air. For the capacitively coupled plasma treatment, the cleaned PI plate was placed on an aluminum flat tray adapted to a cylindrical, stainless steel, low-pressure chamber (diameter 10 cm and depth 27.8 cm), which was evacuated by a rotary vane pump (3.0 m^3^∙h^−1^, DUO 3.0, Pfeiffer Vacuum, Asslar, Germany) in a modular plasma system (Femto SRS, Diener electronic GmbH + Co. KG, Ebhausen, Germany). The system was initiated by a radio frequency generator (13.56 MHz, 0~100 W) to discharge the inlet gas between an aluminum planar electrode and the flat tray under a working pressure of 0.285 torr. The gas flow was manually adjusted by a needle valve (0~50 sccm (standard cubic centimeter per minute), Vögtlin Instruments AG, Aesch, Switzerland), and the chamber pressure was measured by a Pirani sensor (10^−2^~10 mbar). For this initial research, the small-footprint, table-top system was semi-automatically processed through the steps of evacuation pumping, gas inlet, plasma ignition, and ventilation.

The gas (He, N_2_, O_2_, and Ar), RF power (50–90 W), plasma duration (1–3 min), and volume flow rate of the inlet gas (5–30 sccm) were the parameters of the plasma system in the study. Manually matching the forward power to the set power and the reflected power to be less than 20% by adjusting tuning capacitors is required to avoid damaging the generator at each process start and must be sporadically monitored and readjusted during powering on. 

Aliquots (600 μL) of the prepared MIP-QD and NIP-QD solutions were dropped on the center of the plasma-treated PI substrates. The plasma-treated PIs (MIP-plasma-PI and NIP-plasma-PI) were both placed on a hot plate (50 °C) for 15 min. Before baking on a hot plate, coating the aliquots on the PIs with a spin coater (SC-80R, YOTEC, Taiwan) was also tried.

#### 2.2.3. Stripping

The Tc molecules trapped in MIPs were stripped off by immersion of the MIP-plasma-PIs in the solution ethanol/H_2_O = 2 (*v*/*v*) with sonication for 2.0 min. Then, the wet PI plates were spun at 2000 rpm for 30 sec. After repeating the immersion and spin-drying twice, the Tc-stripped PI substrates were ready to probe the Tc. Using different ratios of the stripper EtOH/H_2_O, including 0, 1/2, 1.0, 2.0, and 4.0 as well as 100% EtOH, and dropping the stripper at 5.0 mL/min onto the center of the MIP-plasma-PIs instead of immersion was also tried.

#### 2.2.4. Measurement of Contact Angle and Fluorescence

The sessile drop contact angle and surface free energy of the PI sheets before and after plasma treatments were measured using a contact angle analyzer (100SB, Sindatek, Taiwan) and manually dosing the probe liquids with a 1.000 mL syringe (Hamilton, USA) in a finely adjustable needle holder. The measurement system consisted of a 1280 × 1024 USB 2.0 camera with a fixed focal lens (50 mm, Computar, Japan), a light-emitting diode (LED) backlight, and image analysis software (MagicDroplet). Each PI sample was measured at five locations. Besides, the PIs were characterized by an FTIR spectrometer (Prestige-21, Shimadzu, Japan) equipped with a single reflection horizontal attenuated total reflectance (ATR) accessory (MIRacle, PIKE Technologies, WI, USA).

To load the tetracycline sample (0.56 mM) in phosphate buffer (pH 7.5, 50 mM), two methods, including dropping 10 μL of the sample onto the stripped MIP-plasma-PIs and directly immersing the stripped PIs into the sample solution, were proposed and evaluated by Δ*F* = *F*_0_ − *F*, where *F*_0_ and *F* were the fluorescence intensities measured at 575 nm before and after loading. Here, *F*_0_ was also used to evaluate the previous procedures, including the plasma treatment, the MIP-QDs coating, and the template stripping. After a 2-min equilibration, the sample-loaded PIs were rinsed by pipetting 1.0 mL of the phosphate buffer 5 times, dried by spinning at 2000 rpm for 30 sec, and measured by a spectrofluorophotometer (LS55, Perkin Elmer), which was equipped with a front surface accessory, at an excitation of 315 nm. The measurement processes scheme is in Figure S1.

### 2.3. Selectivity and Stability of the MIP-Plasma-PIs

#### 2.3.1. Imprinting Factors

Prepared at 0.56 mM, the sample solutions of tetracycline; tetracycline analogues including oxytetracycline, doxycycline, minocycline, and methacycline; and steroids including β-estradiol, cholic acid, and hydocortisone were loaded onto NIP-QDs and MIP-QDs on PIs to observe the imprinting factors (IFs). The IF level, defined as Δ*F*_MIP_/Δ*F*_NIP_, where Δ*F* is the change in photoluminescence (PL) intensity (*F*_0_ – *F*) after template binding, demonstrated the superior specificity to the test samples of the imprinted cavities in the MIPs over those of the NIPs.

#### 2.3.2. Recoveries of Tetracycline (Tc) Samples Spiked in Biomatrices

Aliquots of BSA (20 mg∙mL^−1^) or FBS (10% (*v*/*v*)) were added to the Tc solution (5.0 μM) in phosphate buffer (pH 7.5, 50 mM) to simulate the physiological situation and regard as the matrix effect on the recovery percentage (%), which is defined as the ratio of the Δ*F* values measured with the proteins to those without them.

The liquid milk (10 mL) purchased from local supermarkets was spiked with Tc stock solutions to levels of 5.0, 20, and 50 μM. Trichloroacetic acid (0.1% (*w*/*w*)) was then added into the spiked samples, which were sonicated for 10 min, centrifuged at 4000 rpm for 20 min to precipitate the milk protein, and filtered through a 0.22-μm cellulose ester membrane to eliminate particulate matter. The filtrate was adjusted to pH 7.5 with NaOH solution and adjusted to 10 mL with phosphate buffer (pH 7.5, 50 mM).

#### 2.3.3. Storage Stability of MIP-Plasma-PI

A stock solution was prepared by the dissolution of a capsule with 250 mg of tetracycline hydrochloride in phosphate solution (1.0 L, 50 mM, pH 7.5) and the filtration by a 0.22-μm membrane. Parts of the stock solution kept at 4 °C were freshly diluted to 5.0 μM before the recovery measurement of the Tc-stripped MIP-plasma-PIs conditioned in an open-air or nitrogen-filling environment at room temperature. The recovery was collected every third day for one month.

## 3. Results and Discussion

### 3.1. Fabrication of Tc-Templated MIP-QDs on Plasma-Treated PI Substrates

Some significant factors affecting the fabrication and performance of the integrated MIP-QDs on the plasma-treated PI substrates were evaluated and optimized before the analytical determination of the tetracycline target compound in real samples.

#### 3.1.1. Treatments of Plasma on PI Substrates

**Figure 2:**
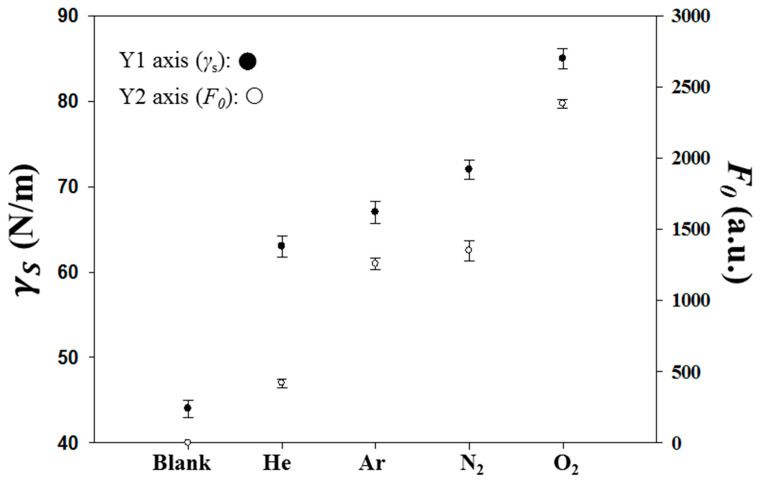
The dependence of various plasma gases on the total surface free energies, $\gamma_{S}$ (●), of the PI substrates and on the fluorescence intensity, F_0_ (○), of the Tc-stripped MIP-plasma-PIs. The plasma was powered at 90 W for 1.0 min with a gas flow of 10 sccm.

First, the type of gas (He, N_2_, O_2_, and Ar) was studied at a fixed volume flow rate of 10 sccm, RF power of 90 W, and duration of 1 min. The surface tension of the plasma-treated PI substrates was measured using the Young–Dupré equation and is plotted against various plasma gases in Figure 2. The equation relates the observed contact angle of a liquid on a surface to the material’s surface free energy, $\gamma_{S}$. Figure 2 shows that plasma treatment increases the $\gamma_{S}$ values, especially for the reactive plasma gases such as N_2_ and O_2_, which chemically implanted in the PI surface and also physically bombarded the surface. The noble gases, He and Ar, were only related to the function of bombardment, and thus, their increases in $\gamma_{S}$ were lower. Without the plasma treatment, the pristine PI substrates do not adhere to the MIP-QD composites. Thus, their F_0_ values, the fluorescence intensities observed at 575 nm with an excitation of 390 nm after both the adhesion of the MIP-QDs and the stripping of the Tc templates off the adhered MIP-QDs, were zero. The O_2_-treated PI substrates possessed the highest $\gamma_{S}$ and F_0_ values among the tested gases, and thus, the O_2_ plasma conditions were further optimized.

**Figure 3:**
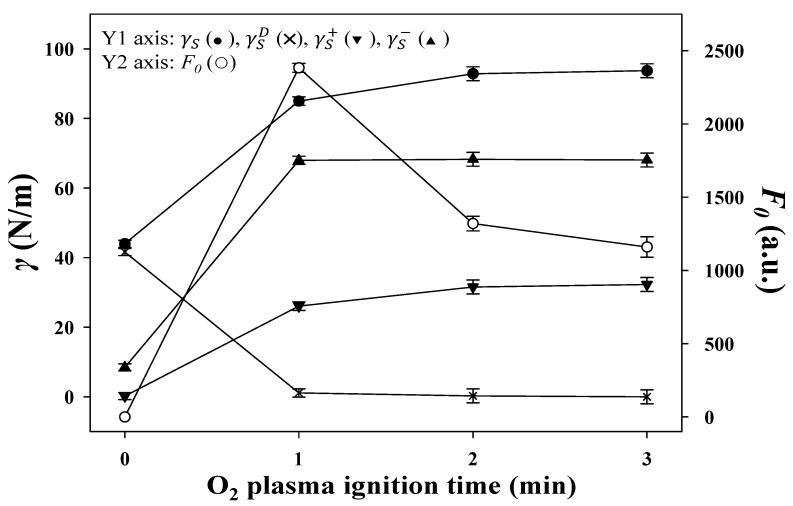
The dependence of O_2_ plasma duration on the total ($\gamma_{S}$, ●), dispersive ($\gamma_{S}^{D}$, **×**), acid ($\gamma_{S}^{+}$, ▼), and base ($\gamma_{S}^{-}$, ▲) surface free energies of PI substrates and on the fluorescence intensity, F_0_ (○), of the Tc-stripped MIP-plasma-PIs. The plasma was powered at 90 W with an O_2_ flow of 10 sccm.

Figure 3 shows the dependence of plasma duration (1–3 min) on the $\gamma_{S}$, $\gamma_{S}^{D}$, $\gamma_{S}^{+}$, $\gamma_{S}^{-}$, and F_0_ values. The $\gamma_{S}$, $\gamma_{S}^{D}$, and $\gamma_{S}^{-}$ parameters are derived from the van Oss–Chaudhury–Good equation [35], which describes the surface energy of a material as being the sum of dispersive, $\gamma_{S}^{D}$, and Lewis acid-base components,$\;\gamma_{S}^{P}$:

(1) $$\gamma_{S}=\gamma_{S}^{D}+\gamma_{S}^{P}$$

where the Lewis acid-base component is defined as:

(2) $$\gamma_{S}^{P}=2{\left(\gamma_{S}^{+}\gamma_{S}^{-}\right)}^{1/2}$$

with $\gamma_{S}^{+}$ and $\gamma_{S}^{-}$ being the acidic and basic contributions to the surface energy, respectively. Thus, the Young–Dupré equation becomes:

(3) $$\frac{1}{2}(1+cos\theta )\;\gamma_{L}={\left(\gamma_{S}^{D\;}\gamma_{L}^{D}\right)}^{1/2}+{\left(\gamma_{S}^{+}\;\gamma_{L}^{-}\right)}^{1/2}+{\left(\gamma_{S}^{-}\gamma_{L}^{+}\right)}^{1/2}$$

**Figure 4:**
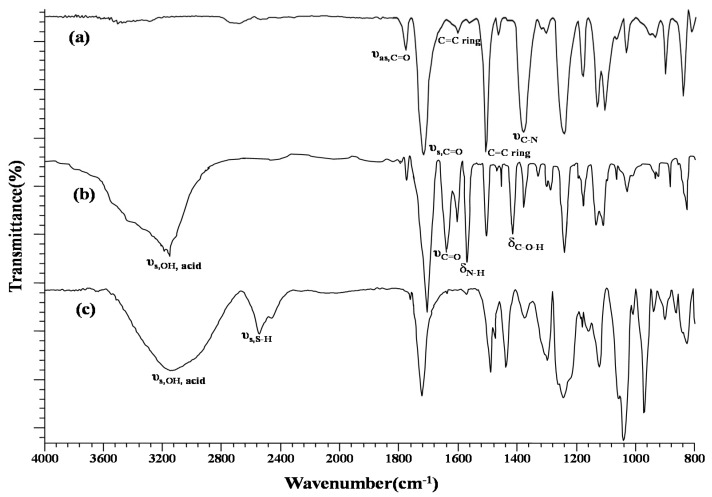
The Fourier transform infrared (FTIR) spectra of (**a**) intact PI, (**b**) plasma-treated PI, and (**c**) MIP-plasma-PI.

Using Equation (3), contact angle measurements were made using three liquids, L (water, ethylene glycol, and glycerol), with known surface tension values, including $\gamma_{L}$, $\gamma_{L}^{D}$, $\gamma_{L}^{+}$, and $\gamma_{L}^{-}$, to determine the individual surface energy contributions, including $\gamma_{S}^{D}$, $\gamma_{S}^{+}$, and $\gamma_{S}^{-}$, for the surface, S, of the PIs. In Figure 3, $\gamma_{S}$ reaches the highest value at 2 min of the O_2_ plasma duration. The $\gamma_{S}^{D}$ values decreased dramatically to near zero and the $\gamma_{S}^{-}$ values rapidly increased to their highest value within 1 min of treatment. The treatment was efficient at incorporating oxygen atoms into the PI surface to increase the surface basicity and thus $\gamma_{S}^{P}$. As shown in Figure 4a,b, the FTIR spectra demonstrate that the incorporation induced cleavage of the imide ring and produced the peaks related to a carboxyl group (υ_s, COOH_ at 3200 cm^−1^ and δ_C-O_ at 1410 cm^−1^) and secondary amide (υ_C=O_ at 1640 cm^−1^ and δ_NH_ at 1570 cm^−1^). Although the PIs treated for 2 min and 3 min possessed higher $\gamma_{S}$ values than those treated for 1 min, their F_0_ values were lower than those of the 1-min treated PIs because the heavy bombardment resulted in rough PI surfaces, subsequent uneven MIP-QD films, and partially peeled MIP-QD coatings by the template stripper, as shown in Figure S2.

The attempt to use O_2_ plasma power lower than 90 W for various times failed, as the resulting $\gamma_{L}$, $\gamma_{L}^{+}$, $\gamma_{L}^{-}$ and F_0_ values were lower than those gained in the 90 W/1 min condition. Due to the 100 W limitation of the RF generator, power higher than 90 W was not tried. The volume flow rate of the gas sources was an insignificant parameter when gas flows faster than 5 sccm were supplied. Therefore, the plasma condition of O_2_/90 W/1 min/10 sccm was applied to the following PIs.

#### 3.1.2. Coating of the Prepared MIP-QDs on PIs

The prepared MIP-QD solution, which had been optimized in the previous study [21], was first spin-coated on the O_2_ plasma-treated PIs. After baking, parts of the coated layer peeled off the PIs while the PIs were immersed in EtOH/H_2_O to strip the Tc template from the MIP network. Although various spin speeds had been tried, the peeling phenomenon persisted. Instead of spinning, simply dripping the MIP-QD solution on PIs ameliorated the peeling problem. This problem may arise from the plasma bombardment and does not occur on the chemically treated PIs and glasses processed by spin coating [21,34]. The comparison of the fabrication and performance of the past and present studies is summarized in Table S1. After many tests, a drip amount of 600 μL was determined as optimal to cover the full plane of PI.

The freshly MIP-QD-coated plasma-treated PIs (MIP-plasma-PIs) were wet and needed to be solidified before treatment with the stripper. Setting the temperature range from 30 °C to 70 °C, both a hot plate and an oven were used to bake the wet PIs for 5~40 min. The highest F_0_ value was found using a 50 °C hot plate for 15 min. As shown in Figure 4c, the characteristic IR absorption peak of S‒H symmetric stretching appearing near 2600 cm^−1^ demonstrated the successful binding of AM, which is the constitutional monomer bridging the MIP and QD on the plasma-treated PIs. The thickness of the coated layer was estimated to be around 100 μm by SEM, which the cross-sectional image shows in Figure 1c and Figure S3. The higher resolved SEM image of the top surface of the layer is also inserted in Figure 1c.

#### 3.1.3. Stripping and Sensing the Tc Template

Higher fluorescence intensity, F_0_, measured after stripping, indicated a higher efficiency of stripping the template quencher from the MIP-QDs. To strip the Tc template molecules from the MIP-QDs on PIs, the entire PI slice was experimentally submerged in the stripper with different volume ratios of EtOH/H_2_O, including 0, 1/2, 1.0, 2.0, and 4.0 as well as 100% EtOH, for two minutes. The highest stripping efficiency was found at the ratio of 2.0, which is the same as in the previous study on the chemically treated PIs with polyacrylate-based MIPs (MIP-chem-PI) [21] but different from another previous study on glass substrates with polysilicate-based MIPs (ratio of 1.0) [34], as summarized in Table S1. The optimal composition of the stripper was determined by the materials of the substrate and the MIP polymer, as the same template was applied. Namely, a hydrophobic stripper is suited to a hydrophobic MIP and substrate. Instead of immersion, dripping the stripper (EtOH/H_2_O = 2.0 (*v*/*v*)) at 5 mL∙min^−1^ on the MIP-plasma-PI center for two minutes did not improve the efficiency. Wetting the complete surface area of the PIs in the stripper improved the release of Tc from the MIP cavities via producing wider and more abundant diffusion paths compared with dripping the stripper onto a confined area. A cyclic repetition of the two steps, stripping immersion for 2.0 min and spin-drying at 2000 rpm for 30 sec, improved the stripping efficiency. However, two repeats were adequate, as further repetition did not grant greater improvement.

**Figure 5:**
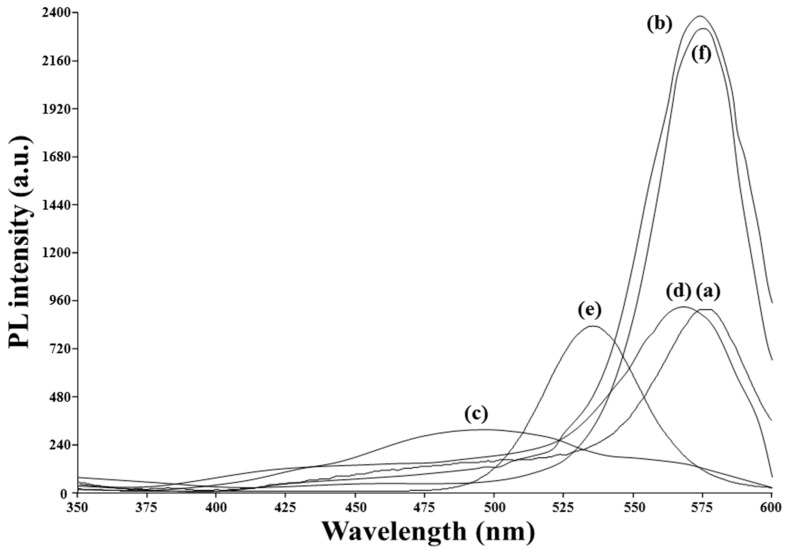
Fluorescence spectra of (**a**) MIP-plasma-PI, (**b**) Tc-stripped MIP-plasma-PI, (**c**) MIP-chem-PI, (**d**) Tc-stripped MIP-chem-PI, (**e**) CdTe QDs, and (**f**) NIP-plasma-PI. Excitation was set at 315 nm. Subfigures (**c**–**e**) were used in ref. [21].

The fluorescence spectra of the MIP-plasma-PIs prepared before and after stripping off the template are shown in Figure 5a,b, respectively. Their peak heights are both located at 575 nm and clearly show the quenching effect from the inclusion of Tc templates in the MIP network. For the MIP-QD-coated chemical-treated PIs (MIP-chem-PIs) prepared before stripping, the 485 nm peak appearing in the spectrum, as shown in Figure 5c, arose from some unknown by-products and/or impurities during the film synthesis and was eliminated by the subsequent stripping, as shown in Figure 5d [21]. Although the coating material used, MIP-QD, was identical for the spin-coating of MIP-chem-PIs and the dripping MIP-plasma-PIs, the applied volume of MIP-QDs on the chemically treated (300 μL) and plasma-treated (600 μL) PIs as well as the baking temperature (65 °C vs. 50 °C) were different. The plasma-treated PIs were cleaner and possessed higher PL intensities, thus facilitating their higher sensitivity to Tc templates than that of the chemically treated PIs. Compared to the original peak of the QDs at approximately 530 nm, as shown in Figure 5e, the peaks appearing at approximately 575 nm in Figure 5 are red-shifted because of the confinement of the QDs and the deep entrapment of some Tc templates in the MIP polymeric network.

The time interval between dropping 10 μL of the 0.56 mM Tc solutions in phosphate buffer (pH 7.5, 50 mM) onto the center of the MIP-plasma-PI sheet and the subsequent rinsing with the buffer accounted for the equilibrium diffusion of Tc into the PI matrix. Two minutes was observed to be sufficient to reach an equilibrium with a stable and relatively high F value (relative standard deviation (RSD) = 2.3%). Although the F values observed at one minute were higher, they were too relatively divergent to be used (RSD = 12.5%). The two-minute equilibrium time was near that of the previous study (3 min) [21] but far from another study (20 min) [34], as summarized in Table S1. The hydrophobic polyacrylate-based MIP and PI substrate helped focus the aqueous Tc solution in the MIP cavities and quickly reach equilibrium. As presented in previous studies [21,34], the immersion of the entire stripped substrates into the Tc solutions was not a better approach to load the sample, as the F values obtained were much lower than those obtained by drop loading.

### 3.2. Fluorescent Measurements of Tetracycline by MIP-QDs on PIs

#### 3.2.1. Imprinting Factors

The fluorescence spectrum of the NIP-plasma-PIs, for which the synthesis did not include the addition of Tc to the porogenic solvent, is shown in Figure 5f. The similarity of the peak positions and intensities between Figure 5f and Figure 5b suggest that the Tc template molecules adsorbed on the MIP-plasma-PIs were stripped thoroughly by the stripper (EtOH/H_2_O = 2.0 (*v*/*v*)). However, the response to the addition of Tc to the NIP-plasma-PIs and MIP-plasma-PIs was different. The difference could be evaluated by imprinting factor (IF), defined as ΔF_MIP_/ΔF_NIP_, to show the imprinted cavities in MIP-QDs possessed better recognizability than the non-imprinted NIP-QDs as they provided adequate dispersion, dipole-dipole, and electrostatic attractions for the template molecules. In the pH 7.5 buffer, the positively charged group of dimethylammonium (pKa ≈9.9) and negatively charged tricarbonyl (pKa ≈3.4) groups of Tc were electrostatically attracted to the negatively charged MAA (pKa ≈4.7) and positively charged AM (pKa ≈10) on the cavity surface. The ΔF values observed for the addition of tetracycline (Tc) template and other samples in 0.56 mM onto MIP-plasma-PI and NIP-plasma-PI plates are shown in Figure S4. For Tc, MIP-plasma-PIs presented a higher IF value (7.2; RSD = 2.2%; n = 5) than MIP-chem-PIs [21] and the suitability for application in a more intricate environment, as summarized in Table S1. Moreover, the imprinted cavities were selective to Tc among its analogues as the IF values of Tc analogues (0.56 mM), i.e., 5.3 of oxytetracycline (RSD = 2.3%), 5.1 of doxycycline (RSD = 2.5%), 3.8 of minocycline (RSD = 2.2%), and 3.4 of methacycline (RSD = 2.2%), were lower than 7.2 of Tc. The selectivity factors, defined as IF_(Tc)_ / IF_(analogue)_ [36], were 1.4, 1.4, 1.9, and 2.1, respectively. Although β-estradiol, cholic acid, and hydrocortisone are also composed of four fused rings, they could not be recognized by the imprinted cavities as they lack the tricarbonyl and dimethylammonium constituents in their structures and their IF values were just near one, and thus their selectivity factors were near to 7.2.

#### 3.2.2. Dose Response

**Figure 6:**
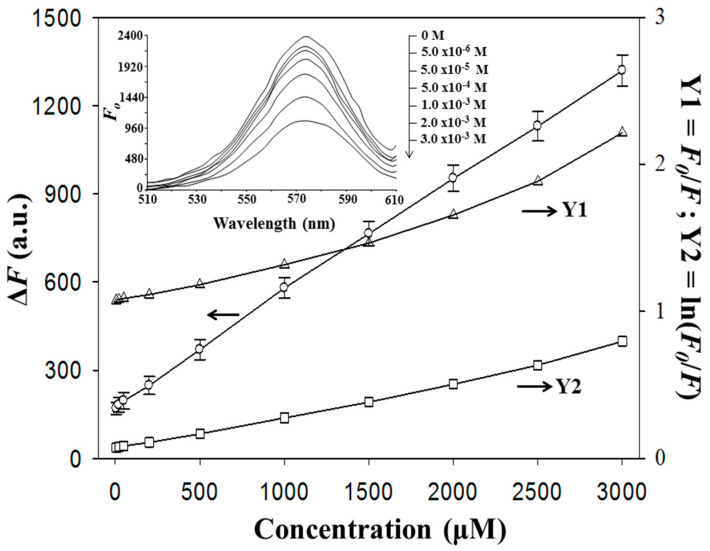
Correlation of the Tc concentration with the (○) Δ F, (Δ) F_0_/F, and (□) ln(F_0_/F) values for the MIP-plasma-PIs in phosphate buffer (pH 7.6, 50 mM). The insert shows the fluorescence spectra at different concentrations of Tc.

The insert of Figure 6 presents the PL intensity decreases as the Tc concentration in the range of 5.0 μM to 3.0 mM increases. The Stern‒Volmer equation, which presents the dynamic collisions between fluorescent and quencher molecules, usually linearly correlates the ratio of PL intensity (F_0_/F) with the quencher concentration [Q]. However, the Stern‒Volmer line sometimes possesses poor linearity (R^2^ = 0.9698), as shown in Figure 6. Although the Perrin model, which combines dynamic quenching with static quenching and proposes linearity between the logarithm of F_0_/F and [Q] [37], was used to correct the linearity to R^2^ = 0.9942, the positive deviation occurring at high Tc concentrations was still not interpreted well because these models are based on the free movement of the QD fluorophores in a solution but not on the confinement of them on PI substrates. Additionally, there may be some surface states existing on PI to help the excited QDs to proceed further with the non-radiation deactivation. Here, alternatively, a better relationship between ΔF and [Q] was introduced (ΔF = 0.3841 ∙ [Q] (Tc concentration at 5.0–3000 μM) + 177.6 (R^2^ = 0.9995)) to attain 0.2 μM of detection limit (3σ, RSD = 2.2%, n = 10). Owing to their brighter PL, the MIP-plasma-PIs were more sensitive to Tc, with a wider linear range and a lower limit of detection (LOD), than the MIP-chem-PIs (range: 70–2200 μM; LOD = 8.8 μM) [21], as summarized in Table S1. The PI plates used, which had been contacted with the sample of 5.0 μM Tc, were tested by the 5.0 μM Tc once again after the stripping process. For ten tests, the average PL intensity was increased by 12 % with a high 15% of RSD.

#### 3.2.3. Detection of Tc in Biomatrices

As discussed for the imprinting factors, MIP-plasma-PI possessed high specificity to Tc over its analogues and steroids in buffers. The addition of protein matrices, BSA and FBS, to the buffers would evaluate the tolerance of the sensory PIs toward biomolecules and add complications to samples. For 300 μg∙mL^−1^ of BSA and 1.5 ppt of FBS used as biomatrices, their recoveries of Tc (5.0 μM) were 98% (n = 5, RSD = 3.2%) and 98% (n = 5, RSD = 2.8%), respectively, as shown in Table 1. The adsorption of Tc on the proteins and/or of the proteins on PI substrates, of which the QD and imprinted cavities would be inhibited, resulted in the recovery deviated from 100%. With more low-molecular-weight molecules, FBS intensified the adsorption strength and thus the recovery deviation more easily than BSA. However, the biomatric effect on the Tc detection on MIP-plasma-PIs was lower than that on the MIP-chem-PIs, for which lower concentration of BSA (μg∙mL^−1^) and FBS (1.0 ppt) were allowed to achieve 97~98% recoveries [21], as summarized in Table S1. Moreover, the variance in the recoveries (RSD = 3.2% (BSA) and 2.8% (FBS)) for the MIP-plasma-PIs was much narrower than that for the MIP-chem-PIs (RSD = 8.2% (BSA) and 9.5% (FBS)) due to the greater consistency in the fabrication of the MIP-plasma-PIs.

The determination of Tc in milk matrices was also studied on the MIP-plasma-PIs. The recoveries of the spiked Tc at 5.0, 20, and 50 μM from milk were 94.5% (RSD = 5.3%, n = 5), 95.2% (RSD = 5.5%, n = 5), and 98.4% (RSD = 6.8%, n = 5), respectively. The deviation of the recoveries from 100% and the resultant RSD levels were attributed to both the pretreatment and measurement procedures. If the Tc standard solutions were spiked in pretreated milk, the recoveries and RSD improved to 97.4% (RSD = 2.5%, n = 5), 97.8% (RSD = 2.4%, n = 5), and 98.7% (RSD = 2.2%, n = 5), respectively, for the spiked concentrations at 5.0, 20, and 50 μM.

#### 3.2.4. Storage of MIP-Plasma-PIs

The recoveries of the Tc drug solutions (5.0 μM) were surveyed every three days over one month to examine the storage stability of the Tc-stripped MIP-plasma-PIs under air and N_2_ surroundings. The preservation in N_2_ maintained average recoveries of 98% (RSD = 2.6%, n = 10) for one month, but the preservation in the air caused a steep rise to 115% on the third day and 123% on the sixth day. The over-estimation of the recoveries was due to the reduced PL intensities induced by oxidation of the QDs on PIs in air. Comparatively, the reduction in PL intensity for the exposure of solid CdTe QD powder to air was much more obvious and rapid within a few hours.

## 4. Conclusions

A comparison of the sensor plates bound with tetracycline-imprinted MIP-QD composites in our series of studies for the fluorometric determination of tetracyclines is summarized in Supplementary Table S1. Among the plates, the MIP-plasma-PI of this study was the most easily fabricated and performed most effectively in the analysis of Tc, exhibiting a wide linear range, low RSD and LOD, and high imprinting factor and tolerance to protein matrices. However, further study of improved preservation in the air over months and multiple reuses is still needed.

## Figures and Tables

**Table 1:** Recoveries (%) of tetracycline (5.0 μM) in phosphate buffer (pH 7.5, 50 mM) containing different concentrations of bovine serum albumin (BSA) and fetal bovine serum (FBS) by fluorescence determination with the MIP-plasma-PIs.

BSA (μg∙mL^−1^)					FBS (ppt, μL∙mL^−1^) **				
100	200	300	400	500	1.0	1.5	2.0	2.5	3.0
99 (2.8) *	98 (3.5)	98 (3.2)	93 (8.6)	89 (9.5)	97 (3.0)	98 (2.8)	93 (3.5)	92 (5.8)	90 (8.5)

* relative standard deviation (RSD, %, n = 5) in parentheses. ** FBS is in solutions containing hemoglobin ≤25 mg∙dL^−1^.

## Supplementary Materials

The following are available online at https://www.mdpi.com/1424-8220/20/9/2723/s1, Figure S1: Fluorescence measurement of Tc samples, Figure S2: Fluorescence measurement of Tc samples, Figure S3: SEM images of the cross-section of the complete MIP-plasma-PI, which thickness is roughly estimated to be around 100 μm, Figure S4: The ΔF values observed for the addition of tetracycline (Tc) template and other samples in 0.56 Mm onto MIP-plasma-PI and NIP-plasma-PI plates. Samples: oxytetracycline (Oxy), doxycycline (Doxy), minocycline (Mino), and methacycline (Meth), β-estradiol (beta-E), cholic acid (CA), and hydocortisone (HC). Table S1: Comparison of the fabrication and analytical performance of the sensor plates bound with tetracycline (Tc)-imprinted MIP-QD composites.
