# Supplementary material for: Effective and Efficient Pretreatment of Polyimide Substrates by Capacitively Coupled Plasma for Coating the Composites of Tetracycline-Imprinted Polymers and Quantum Dots: Comparison with Chemical Pretreatment
Source: Sensors (Basel). 2020 May 10;20(9):2723. doi: 10.3390/s20092723 (PMC7249214; doi:10.3390/s20092723)
Supplement: Supplementary file 1 [file sensors-20-02723-s001.pdf]

Supplementary Information

Effective and efficient pretreatment of polyimide substrates by capacitively coupled plasma for coating the composites of tetracycline-imprinted polymers and quantum dots: Comparison with chemical pretreatment

Ching-Bin Ke [1], Jian-Lian Chen [2, *]

1 Department of Beauty and Health Care, Min-Hwei Junior College of Health Care Management, No.1116, Sec 2, Zhongshan E. Rd., Tainan 73658, Taiwan

2 School of Pharmacy, China Medical University, No. 91 Hsueh-Shih Road, Taichung 40402, Taiwan

* Correspondence: cjl@mail.cmu.edu.tw

Figure S1. Fluorescence measurement of Tc samples.

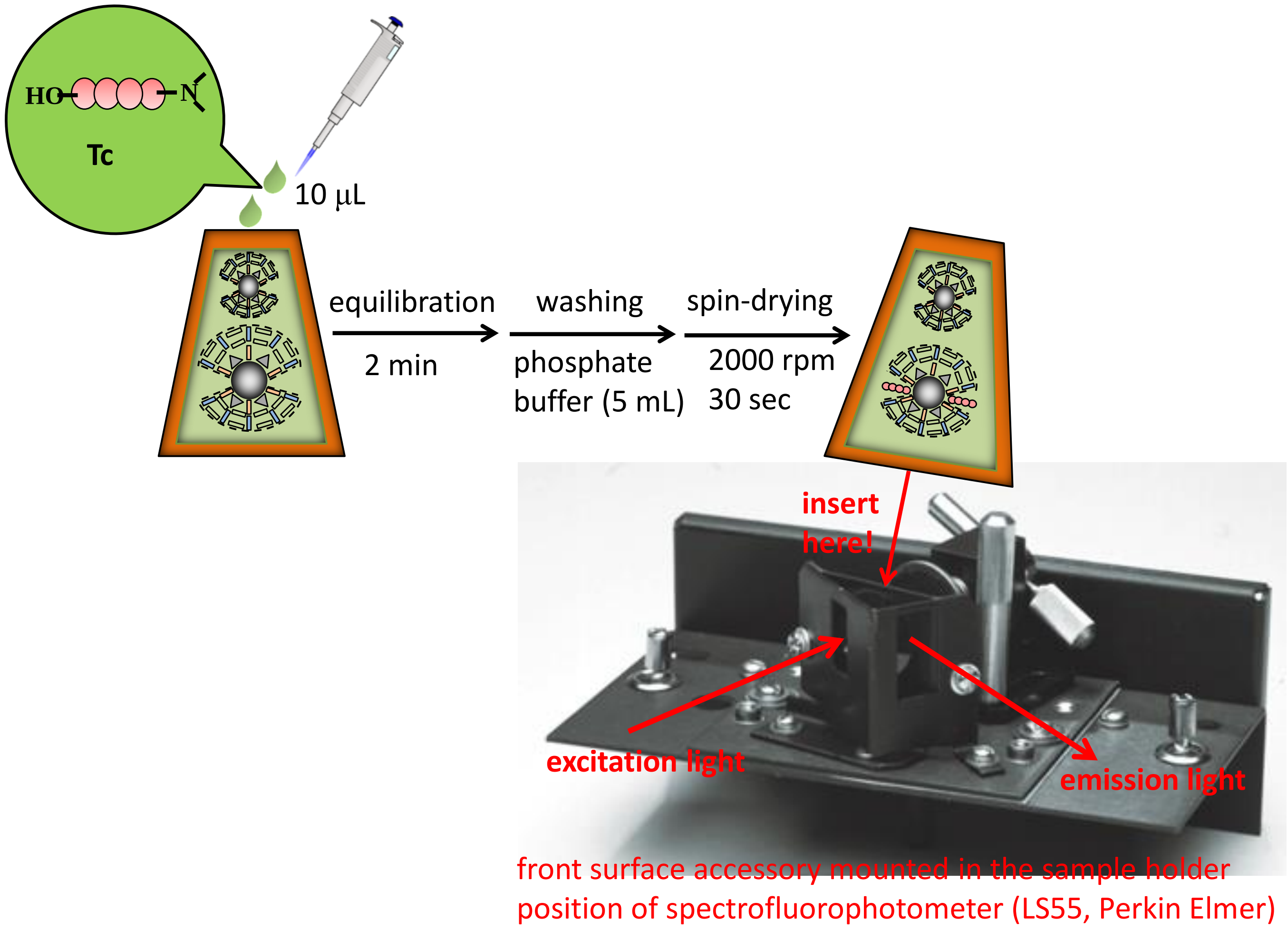

Figure S2. The PIs treated with O_2 plasma for (a) 2 min and (b) 3 min.

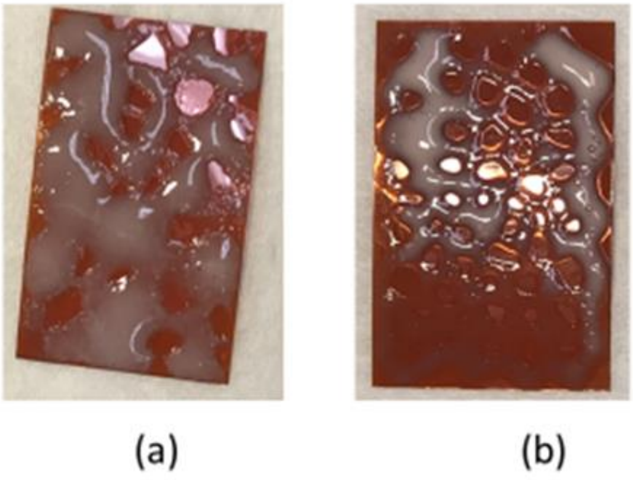

Figure S3. SEM images of the cross-section of the complete MIP-plasma-PI, which thickness is roughly estimated to be around 100 μm.

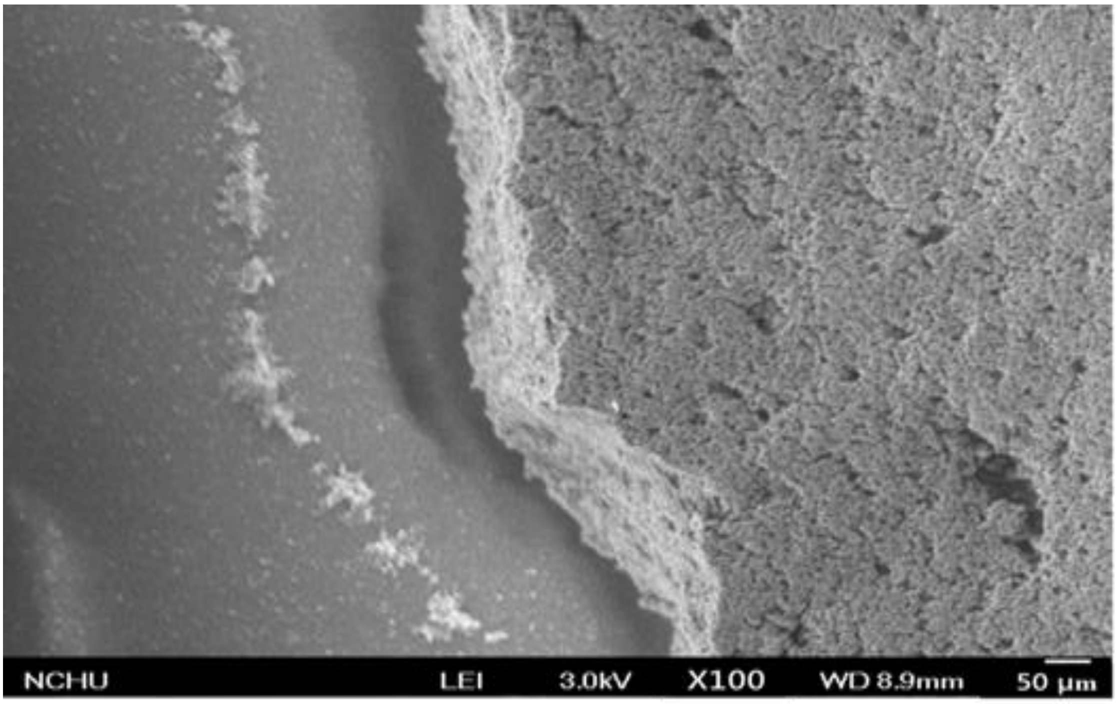

Figure S4. The Δ*F* values observed for the addition of tetracycline (Tc) template and other samples in 0.56 mM onto MIP-plasma-PI and NIP-plasma-PI plates. Samples: oxytetracycline (Oxy), doxycycline (Doxy), minocycline (Mino), and methacycline (Meth), β-estradiol (beta-E), cholic acid (CA), and hydocortisone (HC).

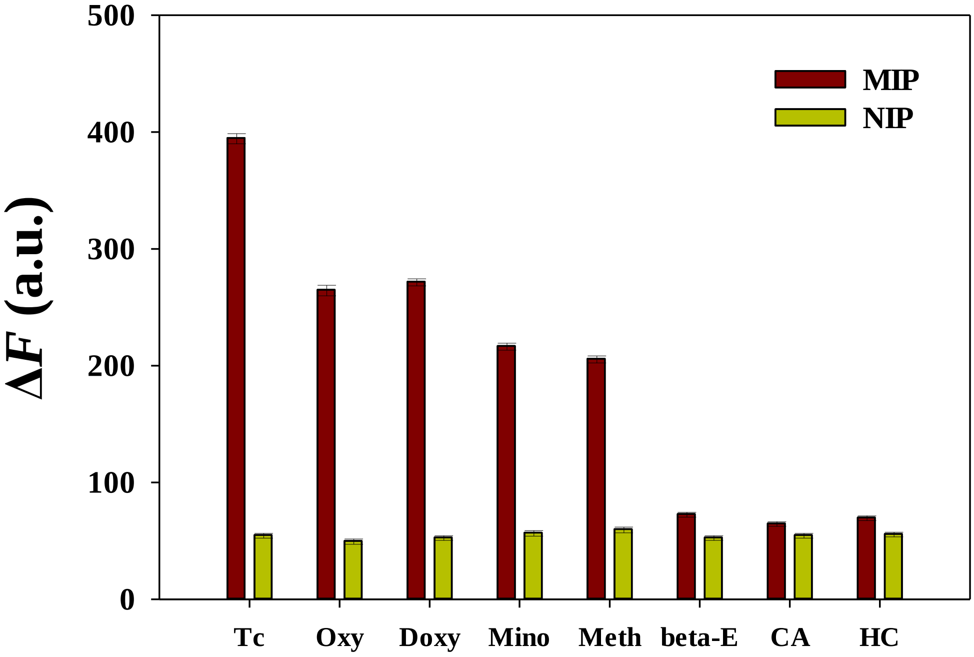

Table S1

Comparison of the fabrication and analytical performance of the sensor plates bound with tetracycline (Tc)-imprinted MIP-QD composites

	MIP-chemical-glass	MIP-chemical-PI	MIP-plasma-PI
MIP bases	Polysilicate; sol-gel polymerization	Polyacrylate; radical-initiated polymerization	Polyacrylate; radical-initiated polymerization
Treated substrates	Silanized glass sheet	Aminolyzed polyimide film	O_2 plasma-treated polyimide film
Bonding of MIP-QDs on substrates	Spin-coating the MIP-QD solution on glass, then baking at r.t. for 6 hours	Spin-coating the MIP-QD solution on PI, then baking at 65 °C for 15 min	Dripping the MIP-QD solution on PI, then baking at 50 °C for 15 min
Stripping Tc template	Immersion in EtOH/H_2O = 1 (v/v) for 1 min	Immersion in EtOH/H_2O = 2 (v/v) for 6 min	Immersion in EtOH/H_2O = 2 (v/v) for 2 min (x3)
Equilibrium time after loading Tc (10 µL) on the sensors	Tc at 0.56 mM for 20 min	Tc at 2.2 mM for 3 min	Tc at 0.56 mM for 2 min
Role of Tc at emission maximum wavelength	Passivator at 560 nm	Quencher at 575 nm	Quencher at 575 nm
Linear relation with the Tc concentration*	$(F–F_0)/I_0$ vs. [Tc] at 70–2200 µM (R^2 = 0.9992)	$(F_0–F)$ vs. [Tc] at 70–2200 µM (R^2 = 0.9993)	$(F_0–F)$ vs. [Tc] at 5.0–3000 µM (R^2 = 0.9995)
RSD; LOD**	RSD = 2.8% (*n* = 10) at 70 µM; LOD = 2.1 µM	RSD = 7.6% (*n* = 10) at 70 µM; LOD = 8.8 µM	RSD = 2.2% (*n* = 10) at 5.0 µM; LOD = 0.2 µM
Imprinting factors***	Tc = 5.6, oxy = 4.4, dox = 3.5, min = 2.6, met = 2.3, steroids ≈ 1	Tc = 4.8, oxy = 4.0, dox = 3.5, min = 3.2, steroids ≈ 1	Tc = 7.2, oxy =5.3, dox = 5.1, min = 3.8, met = 3.4, steroids ≈ 1
The highest tolerance concentrations of BSA and FBS for 97~98% recoveries of spiked Tc****	133 $\mu g \cdot mL^{-1}$ (BSA, 98% recovery, RSD = 3.5%, *n* = 5) and 0.66 ppt (FBS, 97% recovery, RSD = 5.7%, *n* = 5) for spiked Tc (70 µM)	200 $\mu g \cdot mL^{-1}$ (BSA, 98% recovery, RSD = 8.2%, *n* = 5) and 1.00 ppt (FBS, 97% recovery, RSD = 9.5%, *n* = 5) for spiked Tc (70 µM)	300 $\mu g \cdot mL^{-1}$ (BSA, 98% recovery, RSD = 3.2%, *n* = 5) and 1.5 ppt (FBS, 98% recovery, RSD = 2.8%, *n* = 5) for spiked Tc (5.0 µM)
Recoveries of Tc spiking in liquid milk			spiking 5.0 µM Tc in untreated milk: recovery = 94.5% (RSD = 5.3%, *n* = 5); in pretreated milk: recovery = 97.4% (RSD = 2.5%, *n* = 5)
Reference	[34]	[21]	this study

* F_0 and F are the fluorescence intensities measured at the emission maximum wavelength before and after loading Tc.
** LOD is defined as three times the standard deviation of the measurements (n = 10) in the blank solution.
*** Oxytetracycline (oxy), doxycycline (dox), minocycline (min), and methacycline (met).
**** FBS is in solutions containing hemoglobin ≤ 25 mg·dL^{-1}.
